# A novel automated IHC staining system for quality control application in ALK immunohistochemistry testing

**DOI:** 10.3389/pore.2025.1611964

**Published:** 2025-02-13

**Authors:** Chunxiao Hou, Xueru Song, Hongwei Chen, Chengdong Chang, Jinfeng Lu, Cheng Li, Haiyan Qu, Rui Guo, Jingyi Xu, Liming Xu

**Affiliations:** ^1^ Department of Pathology, Haining People’s Hospital, Haining, Zhejiang, China; ^2^ Department of Pathology, The First Affiliated Hospital, Zhejiang University School of Medicine, Hangzhou, China; ^3^ Hangzhou Bailing Biotechnology Co., Ltd., Hangzhou, China

**Keywords:** automated, quality control, anaplastic lymphoma kinase gene ALK, rabbit monoclonal antibody, controls in liquid form (CLFs), lung adenocarcinoma

## Abstract

The establishment of positive and negative controls in immunohistochemistry (IHC) screening for anaplastic lymphoma kinase (ALK) rearrangements is essential in the treatment of lung adenocarcinoma. However, positive control of patient tissue is rare and comes with ethical issues. A novel automated solution for ALK IHC quality control management was investigated by comparison with the established D5F3 antibody on the VENTANA system in 87 lung adenocarcinoma specimens with known ALK status re-analyzed by fluorescence *in situ* hybridization. The BP6165 concentrated antibody on the LYNX480 PLUS platform demonstrated excellent sensitivity and specificity (98.30% and 100%, respectively) in 87 biopsy specimens. The ALK controls in liquid form (CLFs) applied in an automated way showed a more regular circular shape and better cell distribution than those applied manually. In addition, the novel controls can show changes in the same pattern as tissue controls under different antibody concentrations and antigen retrieval conditions. The automated solution for ALK IHC quality control management provides a convenient solution without the consumption of scarce tissue for IHC testing in day-to-day pathology practice. The availability of standardized protocols for the detection of ALK rearrangements using the BP6165 concentrated antibody on the LYNX480 PLUS platform will expand the number of laboratories that can reliably and consistently determine the eligibility of patients with lung adenocarcinoma for treatment with ALK tyrosine kinase inhibitors.

## Introduction

The identification of aberrantly activated tyrosine kinases in a subset of non–small cell lung cancer (NSCLC) has accelerated the approval of ALK tyrosine kinase inhibitors, which have improved the progression-free survival for patients. Approximately 3%–5% of the NSCLC cases harbor an echinoderm microtubule-associated protein-like 4-anaplastic lymphoma kinase (EML4-ALK) rearrangement and may respond to targeted therapy [1–5]. ALK status is clinically pivotal in determining eligibility for ALK-directed targeted therapy. Laboratory tests to detect EML4-ALK rearrangement should be robust, reliable, rapid, and cost–effective. The fluorescence *in situ* hybridization (FISH) method to detect ALK rearrangement was approved by the U.S. Food and Drug Administration (FDA) in 2011 [5–8]. The VENTANA ALK IHC assay, which uses the rabbit monoclonal antibody D5F3 to detect ALK rearrangement, has been authorized by the U.S. FDA as a companion diagnostic for the ALK tyrosine kinase inhibitor Ceritinib [9–12]. Because of the low throughput of FISH, the IHC assay usually plays the role of routine screening of ALK-rearranged NSCLC. The precise detection of low ALK expression mainly depends on the affinity of the antibody, the sensitivity of the detection system, and the quality control [13, 14].

In this study, a novel ALK antibody (clone BP616) performed on an IHC staining system called LYNX480 PLUS was evaluated in a large cohort of NSCLC specimens, to determine its reliability for the detection of ALK rearrangements compared to the D5F3/VENTANA system. The ALK controls in liquid form (CLFs) prepared from genetically modified cell lines applied by the quality control (QC) module of the LYNX480 PLUS have a high-success rate of quality control setting. These novel controls in liquid form could be applied as the quality controls in the IHC staining process to monitor the variation of staining conditions such as antibody dilution rate and antigen retrieval. The auto-QC function of the LYNX480 PLUS combined with the novel CLFs provides a reliable, effective, and donor tissue–saving method of quality control in IHC testing.

## Materials and methods

### Tumor samples and controls

Archival formalin-fixed paraffin-embedded (FFPE) tumor samples from 87 patients with stage I–III NSCLC were retrospectively selected from the Laboratory of Clinical and Experimental Pathology at the First Affiliated Hospital, Zhejiang University School of Medicine between 2021 and 2022. The individual patient data is listed in Supplementary Material S1. In total, 47 ALK-positive specimens and 40 ALK-negative specimens were concurrently confirmed by fluorescence *in situ* hybridization (FISH), qRT-PCR, or IHC in the previous study. All tumor speciments in previous studies were collected, stored, and used with informed written consent of the patients. The study was approved by the local ethics committee (Human Research Ethics Committee, the First Affiliated Hospital, Zhejiang University School of Medicine). An optimized IHC protocol was established by using 3 of the 87 lung adenocarcinoma samples.

### Controls in liquid form (CLFs)

CLFs are a type of cell suspensin prepared from genetically modified cell lines. ALK positive CLF (Catalog Number: BX30026P, Biolynx) and ALK negative CLF (Catalog Number: BX30026N) both were prepared from genetically modified cell lines which could/could not express ALK. CLFs can be applied on slides by pipette or by an automated pipetting system, the QC module in the LYNX480 PLUS while using. The staining pattern and application method for ALK CLFs were validated before use on the 87 tumor samples, by adding CLFs to empty slides and staining these slides with an optimized staining protocol on LYNX480 PLUS. The shape and location of control droplets and the staining patterns of CLFs were compared between automatic and manual applications. The one with better droplet shape, cell distribution, and stable staining patterns was selected as the CLFs application method for further research.

### Automated IHC staining and QC system

The LYNX480 PLUS System (catalog number: I50080B, Biolynx) is an automated immunohistochemistry staining system with a quality control function. The system includes a staining module and an IHC QC module. The IHC quality control module can batch-process 60 slides by adding corresponding CLFs controls before the IHC staining procedure. Automatic cap opening/closing and sampling probe washing can effectively prevent CLFs evaporation and contamination. By scanning the quick response code of CLFs with the scanner in the QC module, the controls in liquid form can be automatically dripped onto the target slides. The applied CLFs droplet can be dried and fixed to the slide in minutes using a heater. Four independent chambers in the staining module can support both IHC and ISH staining simultaneously. The entire QC procedure and information, including patient clinical information, QC, and staining records, can be recorded and tracked.

### Immunohistochemistry

The VENTANA ALK (Clone D5F3) CDx Kit on the VENTANA BenchMark XT platform was performed according to the manufacturer’s recommendations. The rabbit monoclonal anti-ALK antibody BP6165 (catalog number: I1153, Biolynx) assay was performed using the LYNX480 PLUS System with conventional 3,3′-diaminobenzidine (DAB) staining (no amplification). IHC was performed on 3-μm formalin-fixed paraffin-embedded tissue sections.

Preparation steps, which included deparaffinization, rehydration, antigen retrieval, and peroxidase block along with IHC staining processes were all performed on the LYNX 480 PLUS platform with correlated reagents from the BXV visualization system (catalog number: I2003, Biolynx). Antigen retrieval and peroxidase blocking were performed by using the retrieval solution (EDTA) at PH 9.0 for 60 min at 100°C and the peroxidase blocking solution for 5 min. After that, the specimens were incubated with primary antibody (BP6165) for 30 min at room temperature, at the dilution ratio of 1:200. The specimens were then incubated with post-primary antibody for 15 min and secondary antibody-horseradish peroxidase compound for 20 min at room temperature. DAB chromogen concentrate was diluted with DAB diluent at a ratio of 1:20, and the diluted DAB was then applied to each specimen and allowed to react for 10 min. The specimens were then counterstained with hematoxylin. Dehydration was conducted before the specimens were mounted with coverslips. The entire described steps were followed by washes with buffer (TBS) or distilled water. IHC results were evaluated in a blinded manner.

Criteria to assess ALK staining as optimal in the lung adenocarcinoma included: 1) An at least weak to moderate granular cytoplasmic staining reaction of virtually all neoplastic cells in the lung adenocarcinoma with EML-ALK translocation. 2) No staining of neoplastic cells in the lung adenocarcinoma without ALK rearrangement.

### Interpretation of immunohistochemistry staining results

Five independent pathologists from the First Affiliated Hospital, Zhejiang University School of Medicine blindly reviewed each stained slide. Prior to analysis, three lung adenocarcinomas with known ALK status stained with each antibody were reviewed by each pathologist, which allowed them to assess the level of nonspecific or background staining characteristic of the individual reagents. In the vast majority of the tumor cells, the expression of positive ALK protein was defined as that tumor-specific cytoplasmic staining of any intensity was found to be superior to background staining.

### Statistical analysis

Statistical analysis was performed using the SPSS Statistics 22.0 software for Windows (IBM Corporation, NY, United States). Considering how interobserver variability may have impacted the etiologic risk estimates, a Cohen’s Kappa statistic was used to compare the interobserver diagnostic variability among the five different pathologists. A 95% confidence interval (CI) was used to estimate the range of values of a parameter.

## Results

### Clinicopathologic characteristics of patients

Of the 87 patients with lung adenocarcinoma, the median age was 52 years in the current cohort. There were 35 men and 52 women. The pathologic stages were stage I in 40 patients, stage II in 38 patients, and stage III in 9 patients. According to the IALSC/ATS/ERS classification, the most prevalent subtype was acinar adenocarcinoma (37.93%), followed by minimally invasive adenocarcinoma (26.44%), papillary predominant (9.20%), adenocarcinoma *in situ* (6.90%), solid predominant (6.90%), lepidic predominant (6.90%), variants of invasive adenocarcinoma (4.60%) and micropapillary predominant (1.15%). The clinicopathologic characteristics of the patients are listed in Table 1.

**TABLE 1:** Patient clinicopathologic parameters for 87 adenocarcinoma cases.

Characters	Number (%)
Gender	
Male subjects	35 (40.23)
Female subjects	52 (59.77)
Age(years)	
Range	28–74
Median	52
Stage	
I	40 (45.98)
II	38 (43.68)
III	9 (10.34)
ALK status (FISH/qRT-PCR/IHC)	
Positive	47 (54.02)
Negative	40 (45.98)
Adenocarcinoma Subtypes(IASLC/ATC/ERS)	
Adenocarcinoma *in situ*	6 (6.90)
Minimally invasive adenocarcinoma	23 (26.44)
Invasive adenocarcinoma	
Lepidic predominant adenocarcinoma	6 (6.90)
Acinar predominant adenocarcinoma	33 (37.93)
Papillary predominant adenocarcinoma	8 (9.20)
Micropapillary predominant adenocarcinoma	1 (1.15)
Solid predominant adenocarcinoma	6 (6.90)
Variants of invasive adenocarcinoma	4 (4.60)

### Comparison between rabbit monoclonal anti-ALK antibody BP6165 and ALK (D5F3) CDx Kit

**FIGURE 1:**
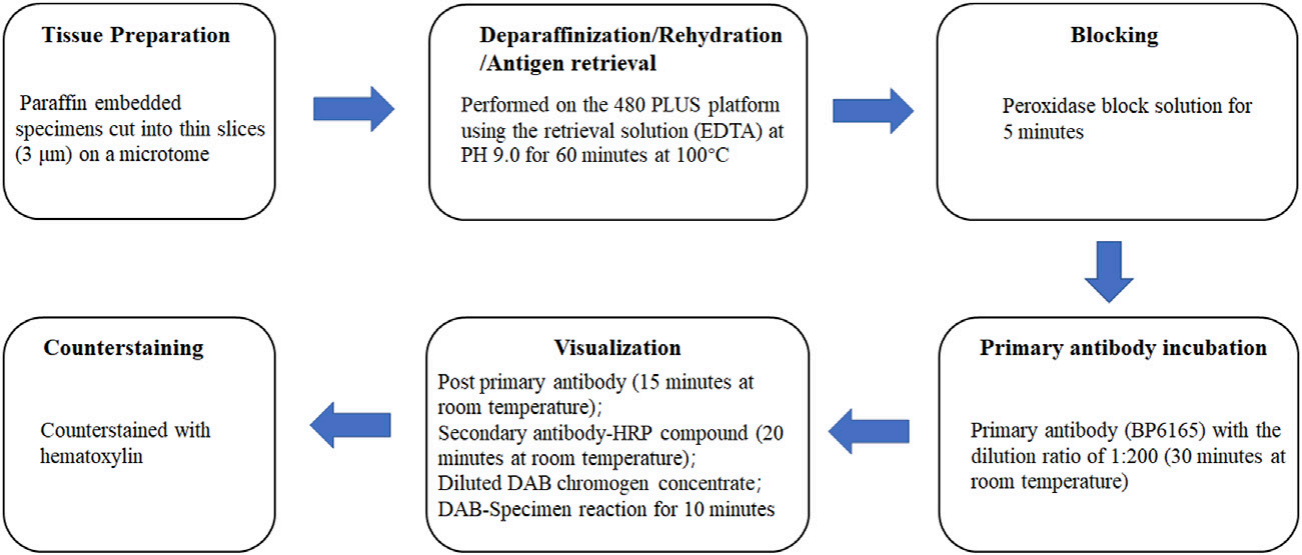
Optimized protocols for ALK IHC assays using the BP6165 concentrated antibody on the LYNX480 PLUS platform.

**FIGURE 2:**
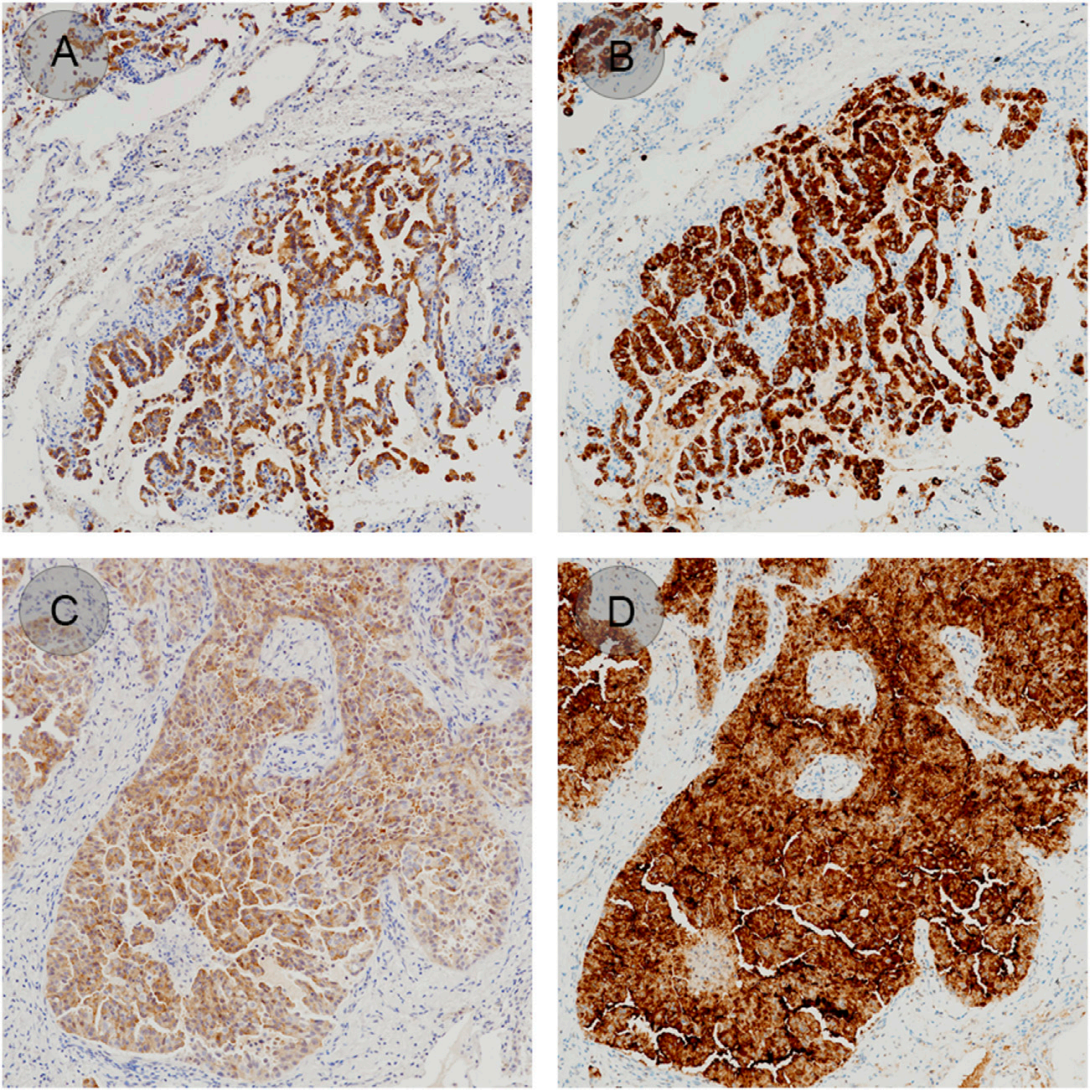
ALK staining patterns on lung adenocarcinoma samples using the BP6165 concentrated antibody on the LYNX480 PLUS platform **(A, C)** compared with the ALK (D5F3) CDx on the VENTANA BenchMark XT platform (gold standard) **(B, D)**.

Three lung adenocarcinoma samples with known EML4-ALK status as determined by qRT-PCR and FISH were analyzed for ALK protein expression using the D5F3/VENTANA and BP6165/LYNX480 PLUS immunohistochemical assays, respectively. According to the optimized protocol (Figure 1) introduced by the manufacturer (Biolynx, China), staining patterns on lung adenocarcinoma samples using the BP6165 concentrated antibody on the LYNX480 PLUS platform showed weaker specific cytoplasmic signals and less background than staining patterns with the ALK (D5F3) CDx Kit on the VENTANA BenchMark XT platform (Figure 2).

### Evaluation of the LYNX480 PLUS platform agreement versus the gold standard

A total of 87 lung adenocarcinoma samples were then used to study the concordance of the BP6165 antibody assay on the LYNX480 PLUS platform with the D5F3 CDx Kit on the VENTANA BenchMark XT platform. The BP6165 antibody assay identified all 47 ALK-rearranged tumors as assessed by five independent pathologists based on the ALK (D5F3) CDx binary scoring algorithm. The average positive percentage agreement (PPA) was 98.30% (Table 2). The diagnostic results of the two platforms as assessed by five independent pathologists showed good agreement (kappa > 0.80) (Table 2). ALK rearrangement was confirmed by FISH in all tumors and two of the cases with weak staining intensity. Of the 47 ALK-positive cases, 45 were undoubtedly scored as positive. Two of 47 cases were scored as negative with the possible reason that these two cases were weakly expressed around the low limit of detection level, and the BP6165-480 PLUS Assay had weaker staining than the D5F3-VENTANA Assay as mentioned in the ‘Comparison between rabbit monoclonal anti-ALK antibody BP6165 and ALK (D5F3) CDx Kit’ section of the Results section. The diagnostic results as assessed by five independent pathologists are provided in Supplementary Material S2.

**TABLE 2:** Positive percentage agreement (PPA) and negative percentage agreement (NPA) for ALK IHC assays using the BP6165 concentrated antibody on the LYNX480 PLUS platform compared with the ALK (D5F3) CDx Kit on the VENTANA BenchMark XT platform (gold standard).

Antibody and platform (LDT)	Pathologist	VENTANA ALK (D5F3) CDx assay	VENTANA ALK (D5F3) CDx assay	Total agreement (95% CI)	Kappa
		NPA (95% CI)	PPA (95% CI)		
BP6165 concentrated antibody on the LYNX480 PLUS	A	100% (91.24%–100%)	100% (92.44%–100%)	100% (95.78%–100%)	1
	B	100% (91.24%–100%)	97.87% (88.89%–99.62%)	99.93% (93.78%–99.80%)	0.98
	C	100% (91.24%–100%)	95.74% (85.75%–98.83%)	97.87% (92.00%–99.37%)	0.95
	D	100% (91.24%–100%)	100% (92.44%–100%)	100% (95.78%–100%)	1
	E	100% (91.24%–100%)	97.87% (88.89%–99.62%)	99.93% (93.78%–99.80%)	0.98
Average		100%	98.30%	99.55%	NA

95% CI: 95% confidence interval.

### CLFs application to slides

**FIGURE 3:**
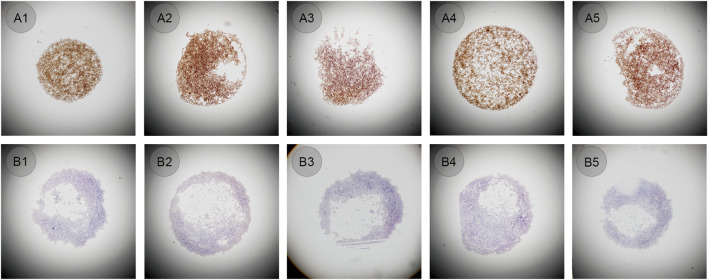
**(A1–A5)** ALK positive controls in liquid form manually pipetted (original magnification: ×4). **(B1–B5)** ALK negative controls in liquid form manually pipetted (original magnification: ×4).

To meet the requirements of setting quality control in ALK testing, CLFs prepared from cell lines was selected and tested to determine whether it could be used as a control. When appropriate IHC protocol was used, 80%–95% of ALK-positive cells were presented as moderate to strong staining on the cytoplasm, while no positive staining was present on ALK-negative cells---this pattern was defined as the standard staining pattern for ALK CLFs defined by the manufacturer. Both ALK-positive cells and negative CLFs were vortexed for 10–15 s and then 1–2 μL were manually applied to the slide by five technicians. All staining showed the same pattern according to the manufacturer’s interpretation guidelines, in which the positive showed 80%–95% cytoplasm staining and the negative showed no positive staining (Figure 3). Cellular structures such as cell membranes, cytoplasmic, and nuclear details, were also well preserved. However, the shape of all the droplets was very different from one to another, as shown in Figure 3, only A1 and A4 are in circle shape but still with different diameters. The cell distribution of some droplets was also found to be uneven, especially in negative cell droplets.

### Comparison of automated and manual application of CLFs

**FIGURE 4:**
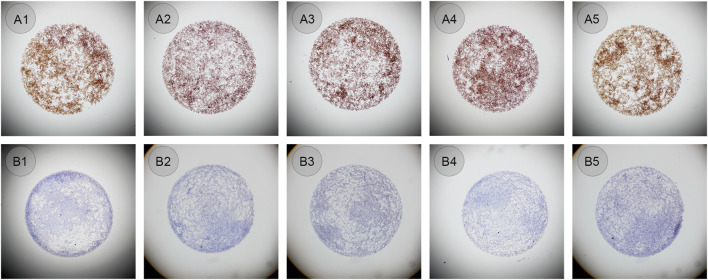
**(A1–A5)** ALK positive controls in liquid form automatically added on the LYNX480 PLUS platform (original magnification: ×4). **(B1–B5)** ALK negative controls in liquid form automatically added on the LYNX480 PLUS platform (original magnification: ×4).

The same bottles of ALK CLFs were performed in an automated way on the QC module in LYNX480 PLUS platform to see whether the droplets shape and diameter could be similar and cell distribution could be even. The negative or positive CLFs was mixed and added automatically by the LYNX480 PLUS to five slides. The staining results showed the standard staining pattern of ALK CLFs. Besides, compared to the manual application, the control droplet applied by the automated method had a more regular circular shape and better cell distribution, as shown in Figure 4. The reason might be that, as an automated instrument LYNX480 PLUS’s sample adding probe could give a consistent liquid adding force and adding speed, so the CLFs droplet’s diameter and the cell density in each droplet can be highly uniform. Therefore, automatic application by the LYNX480 PLUS platform might be an accurate and labor-saving way to add CLFs.

### Determination of the CLFs setting scheme on the LYNX480 PLUS system

**FIGURE 5:**
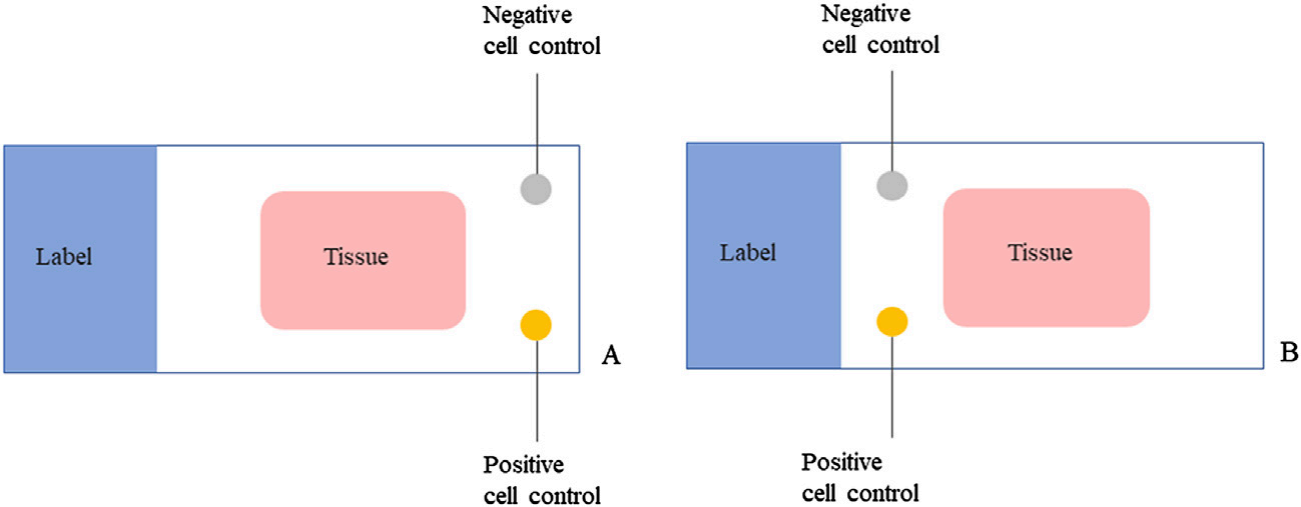
The controls in liquid form setting schemes on the LYNX480 PLUS platform. **(A)** Setting one positive and one negative control in liquid form away from the label end of the slide; **(B)** Setting one positive and one negative control in liquid form near the label end of the slide.

We also tested two control setting schemes to evaluate whether the different setting positions of CLFs could influence the staining results and to determine the better application area on the slide. One was setting the CLFs away from the label end of the slide (Figure 5A). The other was near the label (Figure 5B). After mounting the tissue sections and baking the slides, we applied these two schemes at the same time to the 87 lung adenocarcinoma sample slides using the LYNX480 PLUS platform. The CLFs PPA and NPA results were calculated in Table 3, based on the standard staining pattern of ALK CLFs described in *Results Section* of the Results section. When the controls were set near the label end, the negative percentage agreement (NPA) was 98.8% while the PPA reached 100%, with a total agreement of 99.4%. However, it was only 89.7% for NPA and 90.8% for PPA when the controls were set far away from the label end, with a total agreement of 90.2%, significantly lower than the other control setting scheme. The results of IHC staining are shown in Supplementary Material S3.

**TABLE 3:** Positive percentage agreement (PPA) and negative percentage agreement (NPA) for controls in liquid form set at two different positions on the slide.

Setting the position of controls in liquid form	NPA	PPA	Total agreement
Set near label end of the slide	97.5% (39/40)	100.00% (47/47)	98.85% (86/87)
Set away from the label end of the slide	90% (36/40)	91.49% (43/47)	90.80% (79/87)

After observation, we found that on some slides, the control droplets placed away from the label end were uneven. The reason for this was probably that, the gravitationally-guided melting wax during the slide baking procedure may have flowed through part of the slide space away from the label end. A control droplet was then added there, partially covering the re-solidified wax and affecting the droplet shape during the dewaxing procedure. The same phenomenon was not observed when the controls were set near the label end of the slide. Therefore, we will apply CLFs near the label end for our further study.

### CLFs staining pattern changes in IHC

The processing time of antigen retrieval and the dilution of antibodies could be the main factors influencing the results of ALK staining. Whether CLFs could detect the variation of antigen retrieval and antibody dilution in IHC was verified on the LYNX480 PLUS platform. The confirmed EML4-ALK lung adenocarcinoma samples were used as tissue controls containing positive tumor cells negative lymphocytes and interstitial fiber cells. It was also tested whether CLFs could play the same quality control role as tissue control.

**FIGURE 6:**
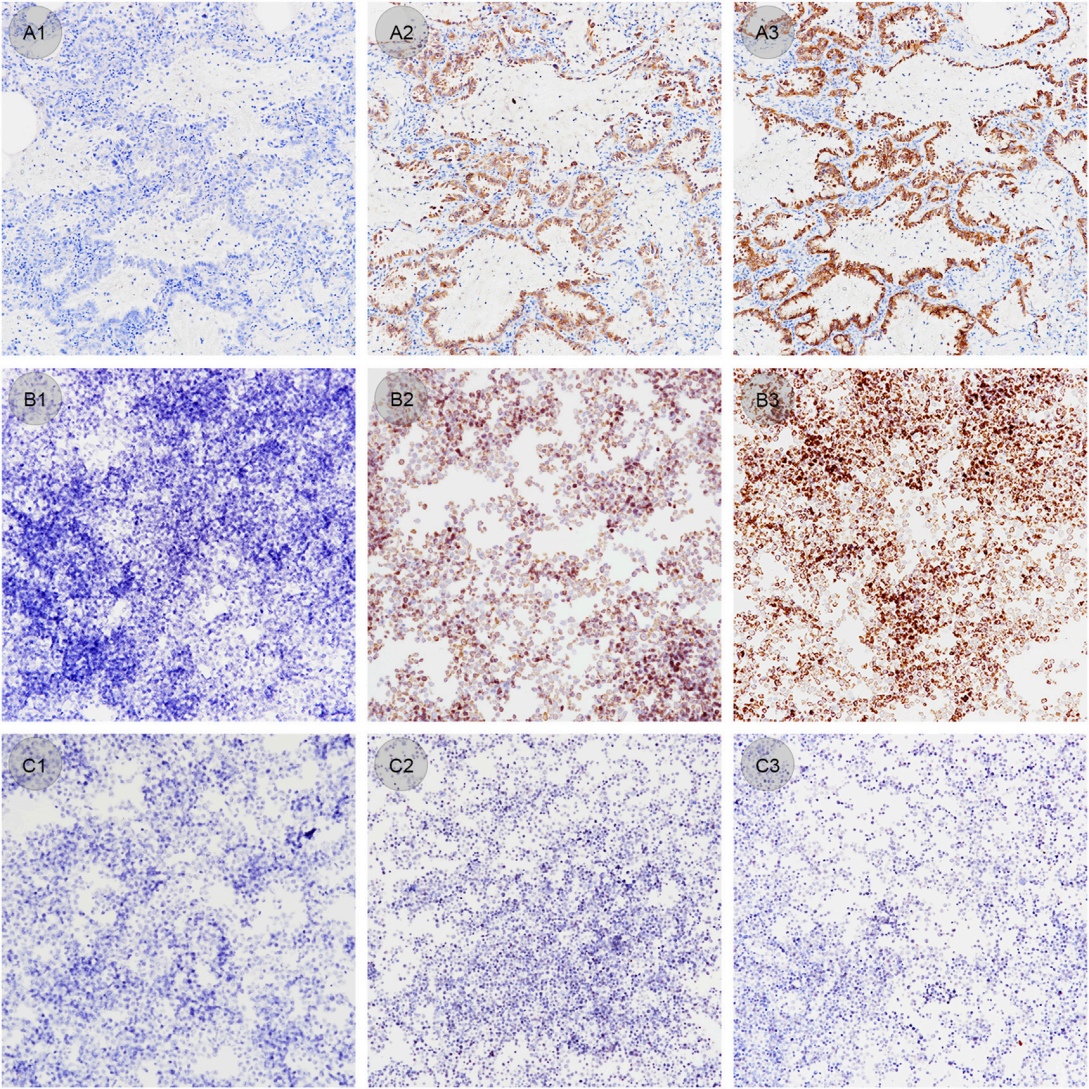
**(A1–A3)** Lung adenocarcinoma samples stained under different antigen retrieval conditions (original magnification: ×20). **(A1)** Antigen retrieval time of 0 min shows a negative staining result. **(A2)** Antigen retrieval time of 20 min shows a weak staining result. **(A3)** Antigen retrieval time of 60 min shows an optimal staining result. **(B1–B3)** ALK positive controls in liquid form stained under different antigen retrieval conditions (original magnification: ×20). **(B1)** Antigen retrieval time of 0 min shows a negative staining result. **(B2)** Antigen retrieval time of 20 min shows a weak staining result. **(B3)** Antigen retrieval time of 60 min shows optimal staining result. **(C1–C3)** ALK negative controls in liquid form stained under different antigen retrieval conditions (original magnification: ×20). Antigen retrieval times of 0 min **(C1)**, 20 min **(C2)** and 60 min **(C3)** all show standard negative staining results.

ALK-positive and negative CLFs were applied simultaneously to 15 slides by an automatic staining device before antigen retrieval. Each slide contained both CLFs droplets and a confirmed EML4-ALK tissue specimen. Staining of CLFs and tissue on five slides treated with proper antigen retrieval time (60 min) showed the standard IHC staining pattern (Figures 6A3–C3). Without antigen retrieval or insufficient treating time (20 min used in this study), positive CLFs showed negative staining or only weak cytoplasmic staining, which was consistent with the staining results of the control tissue specimen (Figures 6A1–C1, A2–C2). Therefore, CLFs can be a quality control material to monitor the effect of antigen retrieval.

**FIGURE 7:**
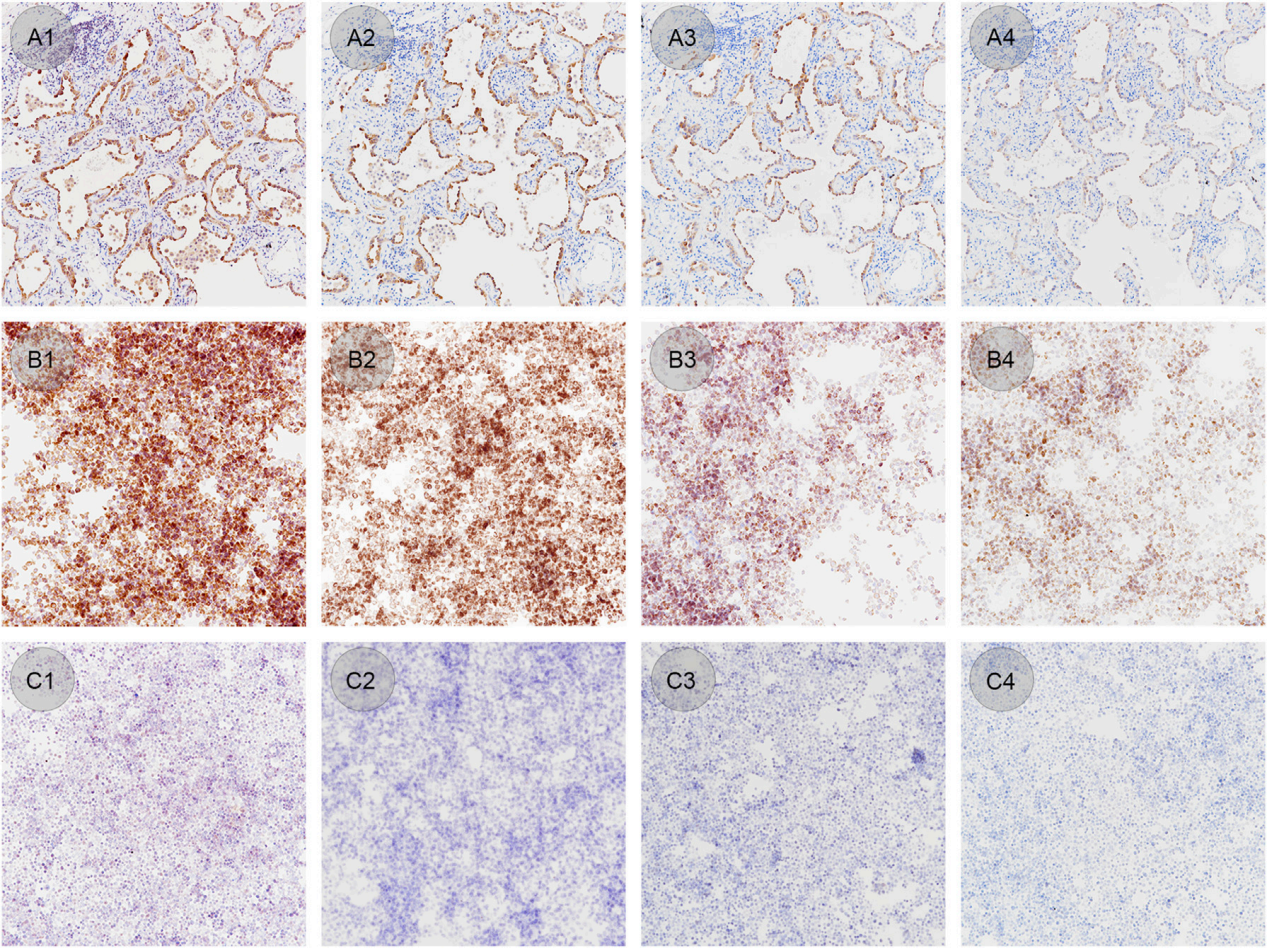
IHC staining results of lung adenocarcinoma samples, ALK positive and negative cell controls by applying different antibody concentrations. **(A1–A4)** Lung adenocarcinoma samples stained with BP6165 at different concentrations (original magnification: ×20). **(A1)** The highest concentration with an antibody dilution rate of 1:25 shows nonspecific staining on lymphocytes; **(A2)** antibody dilution rate of 1:200 shows an optimal staining pattern; **(A3)** antibody dilution rate of 1:1,600 shows weaker staining than the optimal staining; **(A4)** dilution rate of 1:6400 shows even weaker staining. **(B1–B4)** ALK positive controls in liquid form stained with BP6165 at different concentrations (original magnification: ×20). **(B1, B2)** Both antibody dilution rates of 1:25 and 1:200 show a standard staining pattern; **(B3)** antibody dilution rate of 1:1,600 shows a lower positive rate, away from the standard staining pattern; **(B4)** antibody dilution rate of 1:6400 shows even weaker staining. **(C1–C4)** ALK negative controls in liquid form stained with BP6165 at different concentrations (original magnification: ×20). **(C1)** antibody dilution rate of 1:25 shows “background staining;” **(C2–C4)** dilution rates of 1:200, 1:1600, and 1:6400 show standard negative results.

CLFs with confirmed EML4-ALK tissue controls were also stained with different concentrations of ALK BP6165 antibody. The optimal dilution of BP6165 tested previously was 1:200. As shown in Figure 7, all lung adenocarcinoma samples, ALK positive and negative cell control showed a standard staining pattern when the antibody dilution rate was 1:200 (Figures 7A2–C2). When a higher concentration antibody with a dilution rate of 1:25 was applied, the lymphocytes in the lung adenocarcinoma samples showed nonspecific staining and negative cell control showed “background staining” (Figures 7A1–C1). When an antibody dilution of 1:1,600 was applied, lung adenocarcinoma samples showed weaker staining and the positive cell control showed a lower positive rate (Figures 7A3–C3). When a 1:6400 dilution ratio of BP6165 was applied, both lung adenocarcinoma samples and the positive cell control showed even weaker staining (Figures 7A4–B4). Therefore, CLFs and lung adenocarcinoma samples have a high consistency of variation when the antibody concentration changes, indicating that CLFs can be a type of quality control material used to monitor the change in antibody concentration.

## Discussion

Given the daily increasing use of IHC staining as a companion diagnostic tool, the use of positive and negative controls for IHC is essential for process standardization and repeatability [15–17]. FFPE tissue samples pre-confirmed for the presence or absence of specific target antigens have been widely used as positive and negative controls [18, 19]. The preparation of tissue controls has doubled the IHC workload, which is both time-consuming and tissue material-intensive. Moreover, ALK-positive lung adenocarcinoma samples are clinically scarce, making it difficult to meet the demand for an adequate supply of controls.

Here a novel ALK antibody with clone number BP6165 and CLFs produced by genetically modified cell lines and the IHC staining system LYNX480 PLUS with QC setting and recording function was developed. IHC staining with BP6165 antibody was performed by a non-signal-enhancing staining procedure. The staining of lung cancer specimens using the BP6165/LYNX480 PLUS system was highly concordant with the D5F3/VENTANA system. The sensitivity of the BP6165 assay was 98.30%. The specificity of the BP6165/LYNX480 PLUS was 100%. Other studies reported a sensitivity of 95% with 5A4/VENTANA [20], 83%–100% with D5F3/VENTANA [21–23] and 100% with 1A4/VENTANA [12]. The specificity of D5F3/VENTANA and 1A4/VENTANA was reported to be 98% each, respectively [24]. The D5F3/VENTANA assay produced a more intense cytoplasmic signal than BP6165 but with higher background and focal staining. These results may be due to the tyramide signal amplification step in the D5F3/VENTANA system, which requires trained staining and experienced evaluation.

CLFs were successfully applied by the LYNX480 PLUS system, with an increased success rate of ALK-positive control setting when compared to that of tissue control, which is free of tissue consumption and has improved efficiency. The benefits of an automated IHC Staining System with QC function include avoiding human operation error and spontaneous QC operation information recording. When conditions such as antibody dilution and antigen retrieval in a IHC testing changed, the staning results of both CLFs and tissue samples would change accordingly, for both form of IHC controls the changes in staining intensity and positive rate were varied identically. The limitation of this approach is that pathologists may not be familiar with CLFs without tissue morphology. This will limit the application of the new controls in IHC testing. Moreover, to test whether ALK CLFs could work well under more staining conditions, a larger scale of tests using different antibodies and staining platforms is needed. These results suggest that CLFs can be an alternative to traditional ALK tissue quality control, and is reliable, convenient in operation, and saves tissue material, which can further promote the success rate of IHC QC setting and improve the productivity of pathology laboratories.

The availability of standardized protocols for ALK rearrangement detection using the BP6165 concentrated antibody on the LYNX480 PLUS platform will expand the number of laboratories that can determine the eligibility of patients with lung adenocarcinoma for treatment with ALK tyrosine kinase inhibitors in a reliable and concordant manner. Automated application of CLFs via LYNX480 PLUS has a more even distribution than manual application. By comparing the tissue positive control, CLFs can not only monitor the running condition of the IHC staining system and protocol sensitivity but also ensure that the correct antibody and antigen retrieval conditions are applied. The LYNX480 PLUS platform combined with CLFs provides a fast, low-cost solution without the consumption of scarce tissue quality controls for IHC testing in day-to-day pathological practice.

## Data Availability

The raw data supporting the conclusions of this article will be made available by the authors, without undue reservation.
